# Supplementary material for: Extracellular Matrix Components Regulate Bone Sialoprotein Expression in MDA-MB-231 Breast Cancer Cells
Source: Cells. 2021 May 24;10(6):1304. doi: 10.3390/cells10061304 (PMC8225072; doi:10.3390/cells10061304)
Supplement: Supplementary file 1 [file cells-10-01304-s001.zip › cells-1205928-supplementary.pdf]

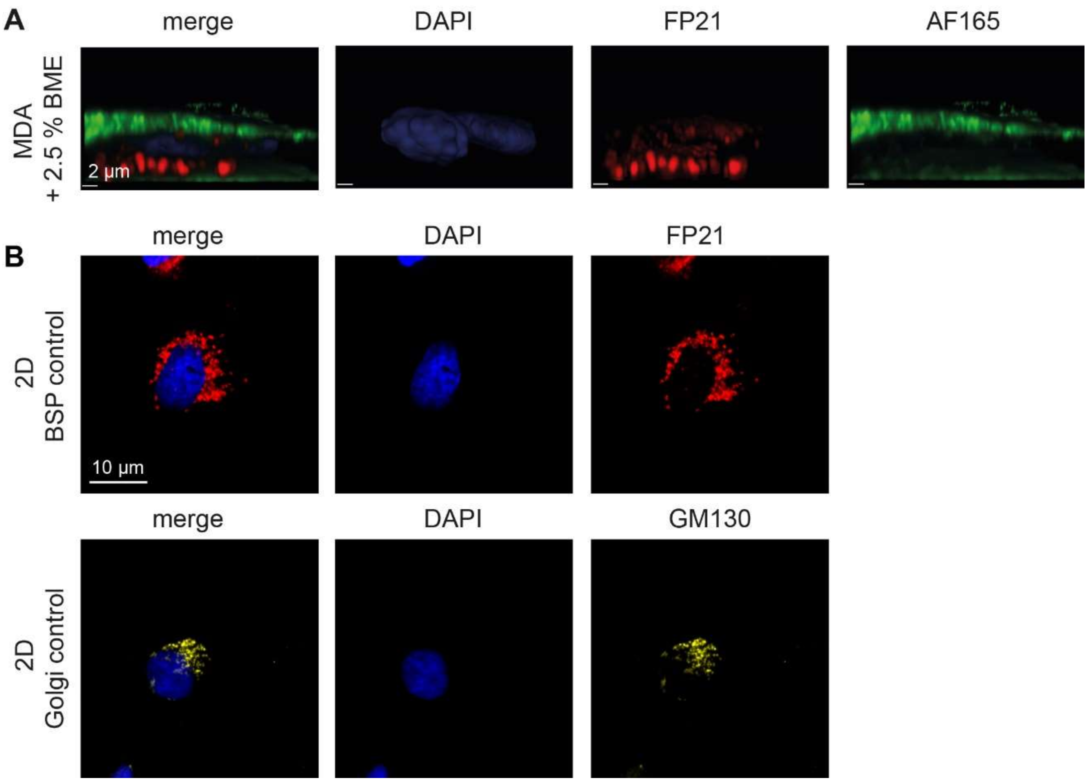

Figure S1: Localization of anti-BSP antibodies AF165 and FP21 in 2D culture. 1,200,000 MDA-MB-231 cells were seeded in petri dishes containing glass cover slips. After 2 days, one culture was supplemented with 2.5 % BME within the media. Alternatively, no BME was added and after 4 days, cultures were PFA fixed and stained for DAPI, human BSP (AF165 and FP21), and GM130 as Golgi marker. A) Exemplary high-resolution side-view of confocal scans for BSP staining in volume projection. B) Representative confocal images in sum projections for direct comparison of FP21 and GM130 signal distribution.

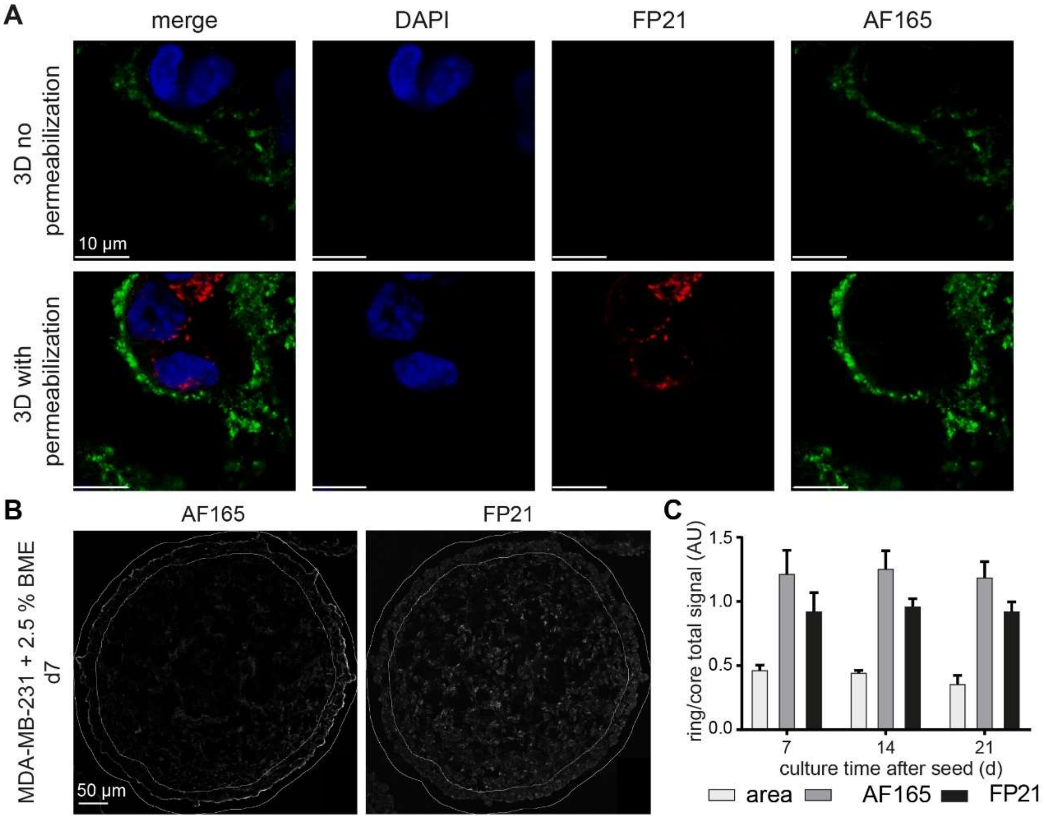

Figure S2: Localization of anti-BSP antibodies AF165 and FP21 in 3D spheroids. 4,000 MDA-MB-231 cells were seeded in mono-culture spheroids and supplemented with 2.5 % BME upon seeding. After 4, 7, 14 and 21 days, spheroids were PFA fixed and stained for DAPI and human BSP (AF165 and FP21) with and without permeabilization as indicated. For spheroid slice quantification, a region of interest on the spheroid rim was set to match around half the total area and to contain roughly the same amount of background regions not containing cells. This ring was then quantified and compared with the corresponding core signal analysis and showed increased AF165 signal in the spheroid rim, while FP21 signal was evenly distributed throughout the investigated time-period A) Exemplary optical slices of whole mount confocal scanned spheroids. B) Representative confocal scans of 10 µm cryo-sectioned spheroids after 7 days in culture with indication of the ring quantification C) Graphs showing quantitative analysis of summed up fluorescence intensities within the outer ring region, normalized with the corresponding core region quantification.
